# Investigating Potential Inhibitory Effect of *Uncaria tomentosa* (Cat's Claw) against the Main Protease 3CL^pro^ of SARS-CoV-2 by Molecular Modeling

**DOI:** 10.1155/2020/4932572

**Published:** 2020-09-30

**Authors:** Andres F. Yepes-Pérez, Oscar Herrera-Calderon, José-Emilio Sánchez-Aparicio, Laura Tiessler-Sala, Jean-Didier Maréchal, Wilson Cardona-G

**Affiliations:** ^1^Chemistry of Colombian Plants, Institute of Chemistry, Faculty of Exact and Natural Sciences, University of Antioquia-UdeA, Calle 70 No. 52-21, A.A 1226, Medellin, Colombia; ^2^Academic Department of Pharmacology, Bromatology and Toxicology, Faculty of Pharmacy and Biochemistry, Universidad Nacional Mayor de San Marcos, Jr Puno 1002, Lima 15001, Peru; ^3^Insilichem, Departament de Química, Universitat Autònoma de Barcelona, Edifici C.n., 08193 Cerdanyola del Vallés, Barcelona, Spain

## Abstract

COVID-19 is a disease caused by severe acute respiratory syndrome coronavirus 2. Presently, there is no effective treatment for COVID-19. As part of the worldwide efforts to find efficient therapies and preventions, it has been reported the crystalline structure of the SARS-CoV-2 main protease M^pro^ (also called 3CL^pro^) bound to a synthetic inhibitor, which represents a major druggable target. The druggability of M^pro^ could be used for discovering drugs to treat COVID-19. A multilevel computational study was carried out to evaluate the potential antiviral properties of the components of the medicinal herb *Uncaria tomentosa* (Cat's claw), focusing on the inhibition of M^pro^. The *in silico* approach starts with protein-ligand docking of 26 Cat's claw key components, followed by ligand pathway calculations, molecular dynamics simulations, and MM-GBSA calculation of the free energy of binding for the best docked candidates. The structural bioinformatics approaches led to identification of three bioactive compounds of *Uncaria tomentosa* (speciophylline, cadambine, and proanthocyanidin B2) with potential therapeutic effects by strong interaction with 3CL^pro^. Additionally, *in silico* drug-likeness indices for these components were calculated and showed good predicted therapeutic profiles of these phytochemicals. Our findings suggest the potential effectiveness of Cat's claw as complementary and/or alternative medicine for COVID-19 treatment.

## 1. Introduction

Severe acute respiratory syndrome coronavirus 2 (SARS-CoV-2) is a part of coronavirus (CoV) family and was initially identified in Wuhan, China, at the end of December 2019. COVID-19 is highly contagious and is most frequently transmitted from human to human, spreading the virus easily to other countries in a very short time [1]. According to the last report of the World Health Organization (WHO), the severe acute respiratory syndrome (coronavirus disease (COVID-19)) caused by SARS-CoV-2 is considered a pandemic, affecting Asia and Europe with the highest death rate followed by America and other regions, causing serious public health problems and considerable economic losses worldwide [2]. Respiratory viral infections are the frequent causes of morbidity and millions of hospital admissions in developing countries every year [3]. For this reason, the pharmacotherapy based on natural products may be a proper alternative for treating viral diseases. On the other hand, traditional medicine is practiced by native South American inhabitants who know the medicinal properties of many plants from the rainforest [4]. Therefore, many of them are collected by ethnobotanists who investigate their resources as antimicrobial [5] and antitumor agents and being the main source for target selection during scientific investigation on compounds with antiviral activity [6]. The biodiversity of South America countries [7] offers a series of medicinal plants which could combat the symptoms of infections such as coronavirus disease COVID-19.

*Uncaria tomentosa* (Willd. ex Schult.) DC. named Cat's claw (“uña de gato” in Spanish) is a woody vine indigenous to the Peruvian Amazon and other tropical areas of South and Central America and belongs to Rubiaceae family [8]. In Peru, natives from the villages in the region of Chanchamayo and Nevati near Puerto Bermudez, Junin, boil approximately 20 g of sliced root bark in 1 liter of water for 45 min commonly and use it in religious purposes and curatives [9]. This species is marketed as dry and ground material remarkably in recent years, being widely studied in all aspects mainly by chemical (isolated chemical structures) and bioassays *in silico, in vitro, in vivo* and some medical trials. Currently, the raw material of *U. tomentosa* is dispensed in Public Hospitals of the Social Health Insurance (EsSalud-Peru) as Complementary Medicine Service (CMS) [10]. Traditionally, extracts prepared by root and bark decoction are used against several diseases, such as allergies, arthritis, inflammations, rheumatism infections, and cancer [11]. However, its popular use has been widely known worldwide, and analytical tests can be found in the United States Pharmacopeia and dosage forms have been authorized only as dietary supplements. Currently, there are about 34 species of Uncaria, with *Uncaria tomentosa* being the most common species [12].

Bioactive constituents of *U. tomentosa* extracts include proanthocyanidins (proanthocyanidin B2 or epicatechin-(4*β* ⟶ 8)-epicatechin, the main component; proanthocyanidin B4, proanthocyanidin C1, an epicatechin trimer, epiafzelechin-4*β* ⟶ 8-epicatechin, and an epicatechin tetramer) [13], oxindole alkaloids (isopteropodine, pteropodine, rhynchophylline, mytraphylline, speciophylline, uncarine F, and uncarine E) [14], indole alkaloidal glucosides (cadambine, 3-dihydrocadambine, and 3-isodihydrocadambine) [15, 16], quinovic acid glycosides, tannins, polyphenols, catechins, beta-sitosterol, and proteins which individually or synergistically contribute to their therapeutic properties [8, 17–24].

In regard to the antiviral properties of *U. tomentosa*, the alkaloid fraction has been demonstrated to be the most effective on human monocytes infected with dengue virus-2 (DENV) *in vitro* [25]. Another study revealed that only the alkaloidal fraction has inhibitory activity on dengue virus, and the negative effect was observed with the nonalkaloidal fraction [26]. In another study, the antiherpetic activity of *U. tomentosa* seems to be associated with polyphenols or their synergistic effect with pentacyclic oxindole alkaloids or quinovic acid glycosides [27]. *U. tomentosa* hydroethanolic extracts demonstrated a significant *in vitro* inhibitory effect on the replication of herpes simplex virus type 1, and the inhibition of viral attachment in the host cells was characterized as the main mechanism of its antiviral activity [27]. Furthermore, other investigations mentioned immunomodulating activity which includes stimulation of phagocytosis, enhancement of B- and T-lymphocytes, suppression of NF-kappa B, and enhancement of IL-1 and IL-6 [28, 29]. In a Peruvian study on rats, the investigators found that phagocytosis was increased and might act as the potent inhibitor of TNF-*α* [30]. In 2008, a study evidenced a possible drug-drug interaction between Cat's claw and protease inhibitors such as saquinavir, atazanavir, and ritonavir, increasing level of these drugs in plasma [31]. Individuals supplemented with a novel water-soluble extract of *Uncaria tomentosa* (C-Med-100®) showed increased effectiveness of pneumococcal vaccination as a result of an increase the lymphocyte/neutrophil ratios of peripheral blood and a reduced decay in the 12 serotype antibody titer responses to pneumococcal vaccination [32].

With neither drugs nor vaccines approved against SARS-CoV-2 yet, finding strategies to diminish the impact of the pandemic is fundamental. Medicinal herbs and, more particularly, those demonstrating antiviral activities are possible allies in this quest. Their use could slow down the spreading of the disease. Particularly in developing countries, in which the accessibility to these plants is easier and more economically viable, adding these medicinal herbs to the general medical kit may be beneficial. In addition, traditional knowledge of these remedies may reduce possible side effects, allowing them to be implemented with fewer medical risks [33].

On the basis of the aforementioned background of *Uncaria tomentosa* (Cat's claw), this work aims at computationally identifying potential bioactive compounds against COVID-19. It focuses on possible interactions and inhibition of the 3CL^pro^ protease (also called M^pro^). 3CL^pro^ is responsible for 100% of the proteolytic mechanism of the virus and is involved in virulence, infectivity, transcription, and replication cycle of the virus [34, 35]. It has been identified as the main druggable target of SARS-CoV-2 for new antiviral discovery. Moreover, its X-ray structure has been recently released, hence allowing possible computational analysis. In fact, several computational studies have already been undertaken on this system including a long 20 *μ*s molecular dynamics (MD) study and virtual screening of several databases [36, 37].

Here, our study stands on a multilevel computational strategy reminiscent to those applied at the early stage of current state-of-the-art drug discovery pipelines and includes (1) protein-ligand docking of all bioactive compounds of Cat's claw against 3CL^pro^ structure, (2) simulations of the ligand pathway of the best predicted compounds from step 1 to evaluate the convenient entrance mechanism of the compounds to the binding site, (3) molecular dynamics simulation to assess the stability of the best protein-ligand complexes from step 2, (4) calculation of the free energy of binding based on MD postprocessing (MM-GBSA), and (5) calculation of pharmacokinetics parameters for the most qualified compounds resulting from the previous parts of the protocol. The study leads to identification of at least three compounds with potential antiviral activity in Cat's claw-based products to propose the extract of *Uncaria tomentosa* as a rapid phytotherapeutic option for COVID-19.

## 2. Materials and Methods

### 2.1. Protein Structure and Setup

Calculated binding affinity of the main constituents of the Cat's claw extracts (Table 1) was explored against the main protease 3CL^pro^ of SARS-CoV-2 findings, a facile therapeutic option for anti-coronaviral therapy; the crystal structure of the protease 3CL^pro^ was downloaded from the Protein Data Bank (PDB entry code 6LU7) [38]; and all bounded ligands, ions, and solvent molecules were manually removed using the DS Visualizer 2.5 program. For docking studies, the structure of the selected protein was parameterized using AutoDock Tools [38]. Gasteiger partial charges were calculated, and polar hydrogens to facilitate the formation of hydrogen bonds were added.

### 2.2. Ligand Dataset Preparation and Optimization

Ligands used in this study are major components of the Cat's claw extracts, a potent irreversible inhibitor recently reported for COVID-19 virus 3CL^pro^ (namely, N3) [39], and three well-known FDA-approved viral protease inhibitors that may be repurposed to treat COVID-19 [40–46]. The 2D structures of 26 Cat's claw constituents were obtained as mol.2 files from the ZINC database [47]. The resultant compounds were submitted to MarvinSketch 8.3 [48] to correct the protonation states of the ligands at physiological pH 7.4 and its structures were parameterized using AutodockTools to add full hydrogens to the ligands, to assign rotatable bonds, and to compute Gasteiger charges and save the resulting structure in the required format for use with AutoDock. All possible flexible torsions of the ligand molecules were defined using AUTOTORS in PDBAutoDockTools [49] to promote the calculated binding with the SARS-CoV-2 protease structure.

### 2.3. Docking-Based Virtual Screening

Our docking protocol was performed using AutoDock 4.2 with the Lamarkian genetic algorithm and default procedures to dock a flexible ligand to a rigid protein. Docking simulation was carried out on the main protease 3CL^pro^ of the SARS-CoV-2 cleavage site (PDB code: 6LU7), where the enzyme residues are in proximity to the recently reported potent inhibitor, known as N3, which was co-crystallized in complex with the main SARS-CoV-2 protease. Once a potential binding site was identified, 26 compounds which are the major components of the Cat's claw extracts were docked to this enzyme site to determine the most probable and the most energetically favorable binding conformations. To accomplish rigorous docking simulations involving a grid box to the identified catalytic site, Autodock Vina 1.1.2 was used [49]. The exhaustiveness was 20 for each protein-ligand pair (number of internal independent runs). The active site was surrounded by a docking grid of 42 Å^3^ with a grid spacing of 0.375 Å. Affinity scores (in kcal/mol) given by AutoDock Vina for all compounds were obtained and ranked based on the free energy binding theory (more negative value means greater binding affinity). The resulting structures and the binding docking poses were graphically inspected to check the interactions using DS Visualizer 2.5 (http://3dsbiovia.com/products/) or PyMOL Molecular Graphics System 2.0 programs [50].

### 2.4. GPathFinder Calculations

To assess the feasibility of the binding route for the ligand, ten runs of GPathFinder [51] calculations for each ligand-protease complex were performed. The ligand was placed at the position obtained from docking calculations and required to search for a possible unbinding route outside the protease. Full flexibility was allowed for the ligand along the pathway including rotameric states of amino acid side chain and backbone motions based on some previous MD simulations of 10 *µ*s. A clustering was carried out by means of the quality threshold methodology [52] to obtain a pool of 110 representative frames from a 10 *µ*s simulation of the apo structure [53]. This pool of representative frames was used as the possible conformations that the protease could adopt during the ligand transport. Vina score and steric clashes were minimized to obtain a total of 120 solutions for each run. A complete input file to use with version 1.2.1 of the software is provided in Supplementary Materials (available here).

### 2.5. Molecular Dynamics Simulations

All the molecular dynamics (MD) simulations were carried out using the dimeric structure of the COVID main protease available in the PDB [53] (code 6lu7) as a receptor. The best-scored docking positions were used as starting conformations for the ligands. Two ligands (one for each monomer) were placed at symmetric positions of the dimeric structure. The protein was prepared by removing waters and crystallographic small molecules from the PDB structure to have the protease residues free of interaction with the ligands during the simulation. Finally, protons were then added through the algorithm implemented in UCSF Chimera Software [54].

MD simulations were set up with the LEaP, which was instructed to solvate the protein with a cubic box of pre-equilibrated TIP3P water molecules and balance the total charge with Na + ions (ions94.lib library). The AMBER14SB force field [55] was used for the standard residues, while the GAFF force field was adopted for the remaining atoms. The geometries of the ligands were optimized in water solvent (SMD continuum model) at DFT level of theory using Gaussian09 [56]. Geometry and frequency calculations were performed using the B3LYP hybrid functional with 6-31G(d,p) as basis set and included Grimme's dispersion [57]. Atomic charges were computed using the RESP protocol (restrained electrostatic potential) at the same level of theory, and the atom types and force constants and equilibrium parameters were assigned using antechamber and parmchk2 from AmberTools18 [58].

For all the MDs, the solvent and the whole system were sequentially submitted to 3000 energy minimization steps to relax possible steric clashes. Then, thermalization of the system was achieved by increasing the temperature from 100 K up to 300 K. MD simulations under periodic boundary conditions were carried out for 250 ns with the OpenMM engine [59] using OMMProtocol [60]. Three simulations were run in total, considering the following protease-ligand systems: speciophylline, cadambine, and proanthocyanidin B2.

Analysis of the trajectories was carried out by means of CPPTraj implemented in AmberTools18. The MD trajectory was considered converged when a stable exploration of the conformational space was achieved. In particular, a stable conformation or a pool of relative stable conformations visited for a statistically consistent number of times were considered as convergence indicators [47]. Considering the alpha carbons of the protease backbone, RMSD from the minimized structure, all-to-all frames RMSD, and cluster counting analysis were performed. Moreover, to ensure that dynamic transitions occur between different conformations, a principal component analysis (PCA) was carried out by plotting the two principal modes relative to each other.

The distance from the geometric center of the ligand to the center of the binding site was computed along all the trajectories. Alpha carbons of the residues GLY143, CYS145, HIS163, HIS164, GLU166, GLN189, and THR190 were considered to calculate the center of the binding site, and the distance from the crystallographic inhibitor N3 to that binding site center (3.947 Å) was taken as reference for comparison.

### 2.6. Prediction of Drug-Likeness Properties for the Most Docking Promissory Compounds

Drug-likeness prediction along with further ADME properties presents a wide range of opportunities for a rapid new antiviral drug discovery. The drug-like and ADME properties for the most active components of the Cat's claw extracts (constituents having the highest binding affinity) were screened using open-access cheminformatic platforms such as Molinspiration (for molecular weight (MW), rotatable bonds, and polar surface area (PSA) descriptors), ALOGPS 2.1 (for log *P*_o/w_ descriptor), and Pre-ADMET 2.0 to predict four pharmaceutically relevant properties such as intestinal permeability (App. Caco-2), albumin-binding proteins (*K*_HSA_), Madin-Darby Canine Kidney (MDCK Line) cell permeation, and intestinal absorption (%HIA). These parameters establish movement, permeability, absorption, and action of potential drugs [61–64]. The interpretation of both MDCK and Caco-2 permeability using PreADMET [65] is as follows:

1. Permeability lower than 25: low permeability
2. Permeability between 25 and 500: medium permeability
3. Permeability higher than 500: high permeability

## 3. Results and Discussion

### 3.1. Database of Cat's Claw Bioactive Compounds

**Figure 1:**
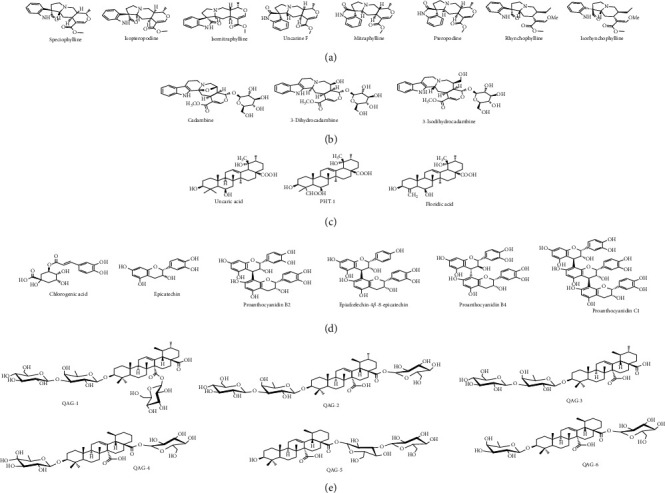
2D structures for the major bioactive constituents of Cat's claw studied as ligands against the SARS-CoV-2 main protease. (a) Spiroxindole alkaloids; (b) indole glycosides alkaloids; (c) polyhydroxylated triterpenes; (d) quinovic acid glycosides.

This study was performed to identify if certain components of the Cat's claw extracts have potential therapeutic effects against COVID-19. To do so, a database of 26 compounds that have shown prevalence on the herbal therapeutic activity has been generated (Figure 1) [8, 9, 13, 23, 25, 29, 66–72]. Our initial hypothesis is that Cat's claw should contain molecules with highest therapeutic profiles against SARS-CoV-2, because of their interaction with 3CL^pro^ main protease.

Currently, some of this chemical components have been isolated and synthesized, such as cadambine [73], speciophylline [74], and proanthocyanidin B2 [75], and several bioassays were performed for their derived products. However, pure drugs that are industrially produced or isolated from plants may be an option for their high biological activity, but they could have some disadvantages. Pure drugs rarely have the same degree of activity as a totalextract at comparable concentrations or doses of the activecomponent [76]. This phenomenon is attributed to theabsence of interacting substances present in the extract.Furthermore, many plants contain chemical constituentsthat could inhibit drug resistance in viral diseases. Synthesizeddrugs are often more expensive to produce, beingunavailable to the poorest populations. In contrast, herbalmedicines used as infusion, decoction, and maceration cansometimes be grown and produced locally, at lower cost,taking into account its ethnopharmacological use.

### 3.2. Docking Results

Despite limitations in terms of energetic functions (scoring) and conformational sampling (limited to ligand rotational bound mainly and restricted local motion of the protein at the most), protein-ligand dockings are still today the main computational strategy to identify potential binders to a given protein target. Protein-ligand dockings of the 26 constituents of Cat's claw were performed against the three-dimensional structure of the SARS-Cov-2 main protease 3CL^pro^ (PDB: 6LU7) [37] to study the potential of ethanolic extracts of Cat's claw for COVID-19 treatment based on its majority of components.

**Figure 2:**
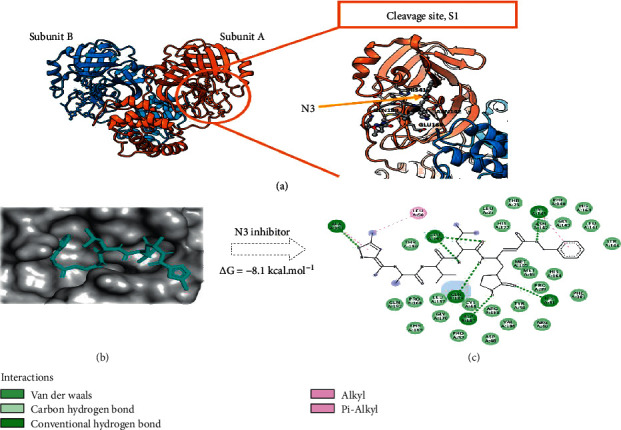
(a) Representation of the 3CL^pro^ protease with N3 inhibitor. Overall structure of the dimer (left) and cleavage site S1. (b) The best conformation of the potent 3CL^pro^ inhibitor N3 into 3CL^pro^ (c) 2D ligand-protease interaction plot between the inhibitor N3 with 3CL^pro^. Dashed lines indicate interactions of N3 with 3CL^pro^.

The structure of 3CL^pro^ shows the protein to be a homodimer with a subunit of 306 amino acids long (Figure 2). The catalytic site is mainly constituted by loops and hairpins that suggests a flexible site, a feature common in many proteases. The co-crystallized N3 drug stands in the catalytic site that defines the subsites of cleavage site S1 and is characterized by the following amino acids: THR190, GLN189, GLU166, HIS164, CYS145, GLY143, GLU166, HIS163, PHE140, ASN142, MET49, HIS41, MET165, ARG188, and ASP187.

This site was defined as the binding pocket for the docking runs. Because of the symmetry in the X-ray structure between the two dimers of 3CL^pro^, dockings were performed on only one monomer. Molecular docking studies were performed with Autodock for all compounds.

**Figure 3:**
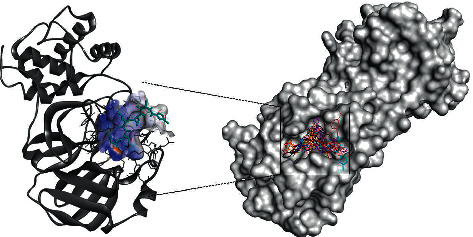
(a) N3 docked into the 3CL^pro^ cleavage pocket. (b) Superposition of the best conformation of the most active components: speciophylline (yellow), uncarine F (blue), cadambine (red), 3-dihydrocadambine (orange), 3-isodihydrocadambine (black), proanthocyanidin B2 (purple), epiafzelechin-4*β*-8-epicatechin (brown), proanthocyanidin B4 (magenta), and N3 (cyan), a potent 3CL^pro^ inhibitor.

All compounds show docked structures that fit well into the S1 cavity of 3CL^pro^ (Figures 3(a) and 3(b)) and with good predicted docking scores that range from −5.9 to −9.2 kcal/mol (Table 1 and Figure S1). In general, calculations revealed that most of the 12 amino acids involved in the N3-3CL^pro^ interactions identified in the X-ray structures also constitute the binding pocket of the docking solutions. In particular, most predicted complexes have the interaction fingerprint with CYS145, GLU166, MET165, GLN189, and GLY143, the most important residues in the reported active pocket for SARS-CoV-2 (Figures 3(b) and 3(c)).

Importantly, some compounds have similar or even lower binding free energy values than those of N3 and other FDA-approved viral protease inhibitors identified as 3CL^pro^ binders which have energies ranging from −8.0 to −8.5 kcal/mol (see the references section of Table 1). At this point, it is worth mentioning that the docking calculations involving these drugs give values in very good agreement with experimental ones and around −8.1 kcal/mol, hence providing a certain amount of confidence regarding the Autodock scoring function of this project.

Notably, nine components have calculated affinities that range from −8.1 to −9.2 kcal·mol^−1^. In this set of nine molecules, we found that most of the proanthocyanidins of the Cat's claw extracts show the higher binding affinities to 3CL^pro^ with regard to any other family of compounds. This result is consistent with several *in vitro* and *in vivo* studies that show that proanthocyanidins display potent virucidal activity for herpes simplex virus (HSV), human immunodeficiency virus (HIV), influenza A and B, hepatitis B and D, human Norovirus, and Aichi virus (AiV) [68, 84]. The five remaining compounds of high affinity for 3CL^pro^ are from the two alkaloidal fractions, the spiroxindole, and the indole glycoside ones. These findings are also consistent with previous reports demonstrating the antiviral properties of the alkaloid fractions from Cat's claw [25]. Amongst those compounds, speciophylline and cadambine are particularly interesting since they are part of the phytochemical fingerprint for *Uncaria tomentosa* (Cat's claw).

From this part of the study, nine components are computationally estimated to have similar or even better binding affinities for the 3CL^pro^ cleavage site S1 than the current protease inhibitor N3 and other antiviral drugs. Speciophylline, cadambine, and proanthocyanidin B2 were selected for further investigation since they are the best representative of three different families of compounds of the Cat's claw fractions (docking scores of −8.1, −8.6, and −9.2 kcal/mol, respectively).

### 3.3. Ligand Pathway Analysis

A first series of refinements consisted in the simulation of the ligand pathways of speciophylline, cadambine, and proanthocyanidin B2 to the catalytic site S1 of 3CL^pro^. Indeed, protein-ligand docking does not account with the protein motions associated to the transition of the ligands from the solvent to the binding site, a phenomenon that could lead to false positives in screening steps of drug discovery processes and to avoid for further stages. To ascertain the accessibility of the compounds to the cleavage site, calculations were performed using the GPathFinder software [51]. For each compound, 120 channels were calculated (10 runs segmented in finding the 12 lowest energy paths), and their results were averaged. The sampling of the protein structure was based on 110 snapshots from the 10 *μ*s simulation of the unbound structure recently released [85], hence allowing that large-scale motions (domain motions) could be accounted in the simulation.

**Figure 4:**
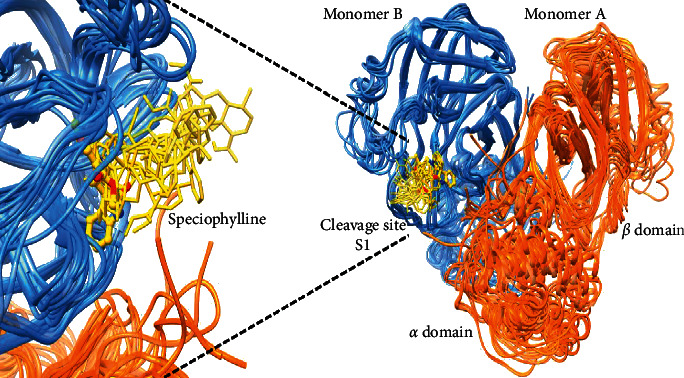
Schematic view of the entrance pathway for speciophylline into monomer B of the 3CL^pro^ protease of SARS-CoV-2. The ligand is shown in yellow and the protein in orange (monomer A) and blue (monomer B). Snapshots of the lowest energy pathway are presented with the ligand represented in thin sticks and the final pose in ball and stick.

For the three compounds, the calculations tend to converge to the same entrance pathway that mainly involve rearrangements of the loop and hairpin motives that constitute the S1 cleavage site as well as some breathing motions involving the alpha helix domain of the dimer (Figure 4). For all ligands, low energy barriers are observed and none overcome the 5.3 kcal/mol in average (±3.2 kcal/mol) (see Table S1). This value is consistent with the relatively solvent exposed binding site of the protease and suggests the absence of restrictive motions for the binding of the ligands. It was noticed that the lowest barrier is observed for speciophylline with a value of 2.4 kcal/mol.

To this point, our study shows that (1) speciophylline, cadambine, and proanthocyanidin B2 (epicatechin-(4*β*-8)-epicatechin) have good predicted binding affinity for S1 cleavage site and (2) they can naturally access it without noticeable energetic cost.

### 3.4. Molecular Dynamics (MD) Simulations and Calculations of the Free Energy of Binding

With higher confidence on the viability of our docking predictions for speciophylline, cadambine, and proanthocyanidin B2, we further evaluated the stability of the docked complexes throughout molecular dynamics simulations. Calculations were performed with openMM software [86] and the Amber force field [55] (for details, readers can refer to Section 2). It is important to notice that the simulations were carried out on the dimeric (hence complete) structure of the protease-ligand complexes after their reconstruction by symmetric operation. For each system, then the stoichiometry of ligand: protein is 2 : 1 (2 ligands for one dimer).

**Figure 5:**
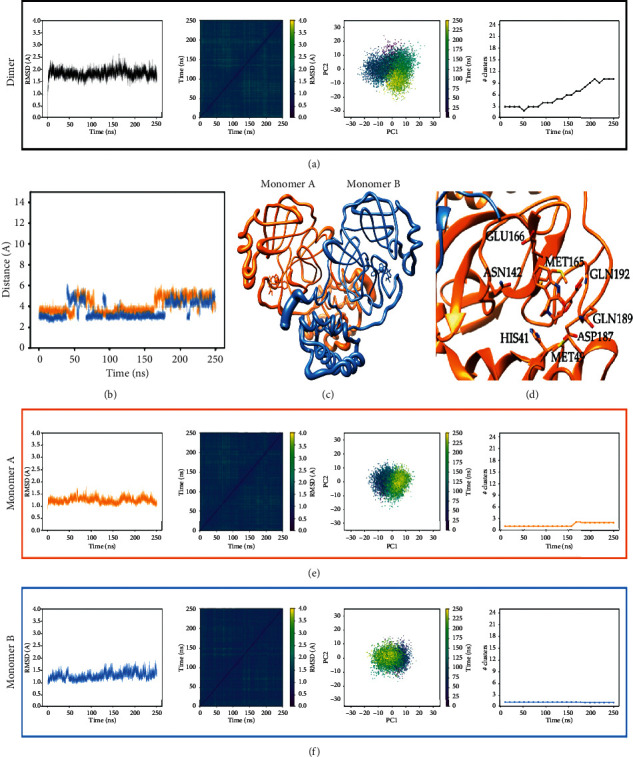
Complete analysis of the 250 ns trajectory of 3CL^pro^ of SARS-CoV-2 bound with speciophylline. Monomer A is shown in orange and monomer B in blue. (a), (e), and (f) shows, from left to right, RMSD, all-to-all RMSD, PCA, and cluster counting (cutoff 1.5 Å). (b) shows the relative position of speciophylline molecules versus the center of mass of the S1 cavity along the simulation (black dashed line indicates the distance of the N3 inhibitor as reference). In (c), size of the ribbon is proportional to the flexibility of the protein backbone and the size on the ligand center is proportional to the amount of structural deviation of the ligand during the 250 ns. (d) reproduces the binding site S1 with the most important residues involved in the interactions.

We assessed the quality of our MD experiment by using four different criteria: (1) root mean square deviation of the backbone (RMSD), (2) all-to-all RMSD, (3) principal component analysis (PCA), and (4) cluster counting. This combination of analytical tools has previously proven to be a reliable assessment methodology of protein structural convergence [87]. Moreover, ligand and protein flexibility were assessed by calculation of RMSF analysis. By taking all these parameters into account, all simulations were run until 250 ns each (see Figure 5 for speciophylline as an illustrative case of analysis, and ESI Figures S3 and S4 for cadambine and proanthocyanidin B2).

Based on the analysis of these different variables and visual inspection, a clear behavior appears along the molecular dynamics, which is summarized as follows:

1. The overall trajectories of all systems are stable with no modification in terms of the secondary structure. Only slight transitions on the tertiary structure (intra- and intermonomeric interfaces) are observed though of different magnitudes depending on which the Cat's claw component is bound and their motion during the course of the MD.
2. For speciophylline, both monomers behave in a very symmetric manner with the ligands remaining well sited into the cavity during the entire course of the simulation and at the same location of the initial docking solution (Figures 5(b) and 5(c)). However, two different orientations are observed. The orientation with higher time of residence is very close to the initial docked complex with most of the interactions of the cleavage site maintained through additional hydrogen bonds that appear with Gln189 and Gln192 (Figure 5(d), Table S2). The second one shows speciophylline displaying a slight rotation around its main axis of inertia (Figure 5(b)). This second orientation appears related to the loss of the hydrogen bond of the Gln189 as well as a slight rearrangement of the *α* domain with respect to the *β* domain. These changes in the ternary structure are also related to modifications of the interfaces between both monomers. It is to note that these motions are consistent with those observed for ligand binding simulation of the previous GPathFinder calculations (see Section 3.3 and Figure 4).
3. For cadambine and proanthocyanidin B2, the overall fold is also very stable. However, a conformational change occurs at about 200 ns, which essentially affects the tertiary and quaternary nature of the system (Figures S3 and S4). Further inspection shows that both monomers have also distinct behaviors. While the ligand remains in monomer B, all along the simulation with poses close to the predicted docked complex and strong interaction with the catalytic binding site, monomer A displaces its ligand at about 200 ns, which then remains closer to the entrance of the monomer-binding site. This displacement toward more solvent exposed location of monomer A is more pronounced for proanthocyanidin B2 than cadambine (Figures S3 and S4). Therefore, cadambine and proanthocyanidin B2 seem to lead to one very stable binding with associated conformational changes that relate, in an apparent allosteric manner, on the second one.

These structural observations were further quantified by MM-GBSA analysis (Table 2). Results show that Δ*G*_binding_ of the species in the different monomers range from ca. −52 kcal/mol to ca. −15 kcal/mol. Taking average values between S1_A_ and S1_B_ sites, the affinity of the different phytochemical compounds to 3CL^pro^ can be classified as cadambine (approx. −40 kcal/mol) > speciophylline (approx. −35 kcal/mol) > proanthocyanidin (approx. −20 kcal/mol). These values suggest the two former molecules to be good binders to 3CL^pro^ and the latest to be somehow of lesser quality. When analyzing each monomer individually, the Δ*G*_binding_ values are consistent with the structural observation with speciophylline having almost symmetric values (ca. −33 kcal/mol) and cadambine and proanthocyanidin having different magnitudes. The closer energy values for cadambine and speciophylline are consistent with the structural similarities between both alkaloids with respect to proanthocyanidin.

In the light of the molecular dynamics analysis and the calculation of the free energy of binding, at least cadambine and speciophylline are predicted to present very good inhibition to the SARS-CoV-2 main protease. Because these components are found in the ethanolic extract of Cat's claw, it may position itself as possible therapeutic herbal for COVID-19.

### 3.5. Calculation of Drug-Likeness Indices and Scoring

Calculated human pharmacokinetics profiles play a critical role in assessing the quality of novel antiviral candidates. Early predictions of pharmacokinetic behavior of the promising antiviral compounds based on their structure could help finding safer and effective leads for preclinical antiviral testing. Herein, we calculated and analyzed various drug-likeness indices for the most qualified Cat's claw components (Table 3). Ten pharmacokinetics parameters were calculated as a drug-likeness filter for speciophylline, cadambine, and proanthocyanidin B2 and compared to N3 and three selected antiviral drugs. Results obtained demonstrate the feasibility of the selected components from Cat's claw exhibiting suitable drug-like characteristics.

In addition, lipophilicity (calculated as log *P*_o/w_) is the key physicochemical property for rational drug design. This parameter provides valuable information about transport through lipid bilayers. Compounds that display high log *P*_o/w_ tend to have good permeability across the cell wall [66, 67]. In this study, the selected compounds exhibited optimal log *P*_o/w_ values < 6.5; notably speciophylline has a log *P*_o/w_ of 1.709 (optimal value compared to 95% of current drugs) implying good permeability across the cell membrane of infected cells. Furthermore, the *in silico* passive transmembrane permeation was calculated for all compounds using Caco-2 cell monolayers or MDCK cells as a model. Cadambine and proanthocyanidin B2 exhibit low values of permeability (<27 nm/s), clearly suggesting poor bioavailability [88]. However, an optimal value for the alkaloid speciophylline was predicted (307 nm/s for Caco-2 model) close to ritonavir and lopinavir drugs [89–91] and was much better than the inhibitor N3 (6 nm/s for Caco-2 model).

Finally, binding to serum albumin (expressed as log *K*_HSA_) is the most important parameter for distribution and transport of antiviral drugs in the systemic circulation. Early prediction of this parameter reduces the amount of wasted time and resources for drug development candidates in the antiviral therapy and management. Behavior of the selected compounds with human plasma protein (log *K*_HSA_) is within recommended values (ranging from −0.30 to −0.044) compared to the reference value taken from 95% of currently known drugs (log *K*_HSA_ from −1.5 to 2.0). Optimal physicochemical properties obtained for the active components of Cat's claw indicated that this herb should be considered for the rapid progression in the antiviral treatment.

## 4. Conclusions

The coronavirus pandemic is a serious public health crisis due to high mortality, high basic reproduction numbers, and neither approved drugs nor vaccines. The recent publication of the crystal structure of the SARS-CoV-2 main protease has provided the community with critical structural information. Potential inhibitors of this enzyme could have a major contribution in the reduction, prevention, or eradication of the viral load of patients.

This study aimed at computationally explore if *Uncaria tomentosa* (Cat's claw), an indigenous medicinal herb known for its antiviral properties against other high mortality viruses, contains phytochemicals potentially able to inhibit the SARS-CoV-2 main protease, 3CL^pro^.

After screening 26 key compounds of *Uncaria tomentosa* against the 3CL^pro^ cleavage site, nine compounds displayed lower binding energies than those of known inhibitors of this enzyme (N3 as well as three FDA-approved antiviral drugs). Amongst those nine compounds, one of each family of well-characterized active phytochemical fractions of Cat's claw, namely, speciophylline, cadambine, and proanthocyanidin B2, attracted our attention because of their strong docking scores (−8.1, −8.6, and −9.2 kcal/mol, respectively). The potential inhibitory effects of those molecules were further analyzed by means of ligand pathway simulations (which show very low barriers for binding), molecular dynamics of the docked complexes (until convergence at aprox. 250 ns), and MM-GBSA free energy binding calculations (with values ranging from ca. −50 kcal/mol to ca. −15 kcal/mol). Altogether, these results confirm that the three compounds, and more particularly the alkaloid ones, have good predicted inhibitory profiles. To anticipate the therapeutic behavior ofCat's claw components, several physicochemical and ADME-score indices were calculated using themore activemolecules and compared to antiviralmarketed drugs. The returned values show optimal drug-like properties for speciophylline, cadambine, and proanthocyanidin B2.

Due to the remarkable presence of these compounds in the Cat's claw extracts, we believe that this *in silico* study at least points at *Uncaria tomentosa* as a whole as an interesting herb opening novel therapeutically horizons for COVID-19 treatment. Based on our findings, we believe that Cat's claw should be taken into consideration in looking for COVID-19 treatments.

## Figures and Tables

**Table 1:** Best binding energy (kcal/mol) based on AutoDock scoring of the main constituents of Cat's claw into the cleavage site of the novel SARS-CoV-2 main protease (PDB ID: 6LU7).

Main constituents of Cat's claw	Best binding energy (kcal/mol)
*Spiroxindole alkaloids*	
Speciophylline	−8.1
Isopteropodine	−6.6
Isomitraphylline	−7.6
Uncarine F	−8.2
Mitraphylline	−7.0
Pteropodine	−7.0
Rhynchophylline	−5.9
Isorhynchophylline	−6.1

*Indole glycoside alkaloids*	
Cadambine	−8.6
3-Dihydrocadambine	−8.0
3-Isodihydrocadambine	−8.0

*Polyhydroxylated triterpenes*	
PHT-1	−6.8
Uncaric acid	−7.0
Floridic acid	−7.6

*Quinovic acid glycosides*	
QAG-1	−7.8
QAG-2	−7.4
QAG-3	−7.2
QAG-4	−7.9
QAG-5	−7.8
QAG-6	−7.8

*Proanthocyanidins*	
Chlorogenic acid	−6.8
Epicatechin	−7.2
Proanthocyanidin B2	−9.2
Epiafzelechin-4*β*-8-epicatechin	−8.9
Proanthocyanidin B4	−9.2
Proanthocyanidin C1	−8.8

*References*	
N3^a^	−8.1 (−8.1)^b^, (−8.3)^c^, (−7.9)^d^
Remdesivir^h^	−8.5
Ritonavir^h^	−8.1 (−7.7)^e^, (−7.5)^f^, (−8.9)^g^
Lopinavir^h^	−8.0 (−8.4)^e^, (−7.4)^f^, (−9.4)^g^

^a^Potent irreversible inhibitor of SARS-CoV-2 virus 3CL^pro^. ^b^Binding affinity reported by [47]. ^c^Binding affinity reported by [77]. ^d^Binding affinity reported by [78]. ^e^Binding affinity reported by [79]. ^f^Binding affinity reported by [80]. ^g^Binding affinity reported by [3]. ^h^FDA-approved antiviral drugs promising to treat SARS-CoV-2 [81–83].

**Table 2:** Ligand-protease Δ*G*_binding_ in kcal/mol.

	Cadambine	Proanthocyanidin B2	Speciophylline
Monomer B	−51.92 ± 6.03	−21.20 ± 6.22	−33.49 ± 4.88
Monomer A	−32.25 ± 5.84	−15.25 ± 7.31	−33.34 ± 6.15

The average obtained from 250 frames for each MD trajectory (one per nanosecond) using the MM-GBSA method in AmberTools18. The following parameters were used: igb = 2, saltcon = 0.100.

**Table 3:** Calculated drug-likeness properties of the most qualified Cat's claw components.

Compound	MW^a^	PSA^b^	n-Rot Bond (0-10)	n-ON (<10)^c^	n-OHNH^d^	Log *P*_o/w_^e^	Log *K*_HSA_^f^	Caco-2^g^ (nm/s)	App. MDCK (nm/s)^h^	% HIA^i^	Lipinski rule of five (≤1)
Speciophylline	368.432	82.804	1	8	1	1.709	−0.044	307	153	81	0
Cadambine	544.557	158.806	8	11	5	0.037	−0.592	27	11	27	0
Proanthocyanidin B2	578.528	209.177	10	12	10	0.505	−0.300	1	1	<25	1
N3	680.800	221.219	17	14	3	2.578	−0.497	6	11	85	2
Remdesivir^j^	602.583	196.086	16	16	5	1.135	−0.685	37	14	36	2
Ritonavir^j^	720.943	139.542	18	11	3	6.335	0.638	647	1014	75	2
Lopinavir^j^	628.810	124.690	16	9	4	5.751	0.554	510	598	83	2

^a^Molecular weight of the hybrid (150–500). ^b^Polar surface area (PSA) (7.0–200 Å^2^). ^c^n-ON number of hydrogen bond acceptors <10. ^d^n-OHNH number of hydrogen bond donors ≤5. ^e^Octanol water partition coefficient (log *P*_o/w_) (–2.0 to 6.5). ^f^Binding-serum albumin (KHSA) (−1.5 to 1.5). ^g^Human intestinal permeation (<25 poor, >500 great). ^h^Madin-Darby canine kidney (MDCK) cell permeation. ^i^Human intestinal absorption (% HIA) (>80% is high, <25% is poor). ^j^FDA-approved antiviral drugs used as references.

## Data Availability

All data used to support the findings of this study can be made available from the corresponding author upon request.

## Abbreviations

PCA: Principal component analysis
ADME: Absorption, distribution, metabolism, and excretion
MD: Molecular dynamics
FDA: Food and Drug Administration
TNF*α*: Tumor necrosis factor alpha
EsSalud: Seguro Social de Salud (in Spanish)
PDB: Protein Data Bank.

## Supplementary Materials

Supplementary Materials Figure S1: histogram showing molecular docking results between the novelSARS-CoV-2 main protease and nine components from Cat's claw which showed the highest binding affinity. Figure S2: (A) the best conformation of the nine active components inside the catalytic pocket of the SARS-CoV-2 main protease 3CL^pro^ (PDB: 6LU7). 2D interaction mode plots of the selected compounds with 3CL^pro^. Interactions between each component and amino acid residues inside the 3CL^pro^ cleavage domain are indicated by the dashed lines. Figure S3: complete analysis of the 250 ns trajectory of 3CL^pro^ ofSARS-CoV-2bound with cadambine. Monomer A is shown in orange and monomer B in blue. Panels A, E, and F show, from left to right, RMSD, all-to-all RMSD, PCA, and cluster counting (cutoff 1.5 Å). Panel B shows the relative position of cadambine molecules versus the center of mass of the S1 cavity along the simulation (black dashed line indicates the distance of the N3 inhibitor as reference). For panel C, size of the ribbon is proportional to the flexibility of the protein backbone and the size on the ligand center is proportional to the amount of structural deviation of the ligand during the 250 ns. Panel D reproduces the binding site S1 with the most important residues involved in the interactions. Figure S4: complete analysis of the 250 ns trajectory of 3CL^pro^ of SARS-CoV-2bound with proanthocyanidin B2. Monomer A is shown in orange and monomer B in blue. Panels A, E, and F shows, from left to right, RMSD, all-to-all RMSD, PCA, and cluster counting (cutoff 1.5 Å). Panel B shows the relative position of proanthocyanidin B2 molecules versus the center of mass of the S1 cavity along the simulation (black dashed line indicates the distance of the N3 inhibitor as reference). For panel C, size of the ribbon is proportional to the flexibility of the protein backbone and the size on the ligand center is proportional to the amount of structural deviation of the ligand during the 250 ns. Panel D reproduces the binding site S1 with the most important residues involved in the interactions. Table S1: GPathFinder vina barriers for the ligand route. 120 solutions were obtained for each ligand. Average and standard deviation are reported. Table S2: amino acids of the 3CL^pro^ protease that participate in hydrogen bonding interactions with cadambine, proanthocyanidin B2, and speciophylline. Input file (.yaml format) used in GpathFinder calculations.
