# Supplementary material for: Investigating Potential Inhibitory Effect of Uncaria tomentosa (Cat's Claw) against the Main Protease 3CLpro of SARS-CoV-2 by Molecular Modeling
Source: Evid Based Complement Alternat Med. 2020 Sep 30;2020:4932572. doi: 10.1155/2020/4932572 (PMC7532411; doi:10.1155/2020/4932572)
Supplement: Supplementary Materials — Figure S1: histogram showing molecular docking results between the novelSARS-CoV-2 main protease and nine components from Cat's claw which showed the highest binding affinity. Figure S2: (A) the best conformation of the nine active components inside the catalytic pocket of the SARS-CoV-2 main protease 3CLpro (PDB: 6LU7). 2D interaction mode plots of the selected compounds with 3CLpro. Interactions between each component and amino acid residues inside the 3CLpro cleavage domain are indicated by the dashed lines. Figure S3: complete analysis of the 250 ns trajectory of 3CLpro ofSARS-CoV-2bound with cadambine. Monomer A is shown in orange and monomer B in blue. Panels A, E, and F show, from left to right, RMSD, all-to-all RMSD, PCA, and cluster counting (cutoff 1.5 Å). Panel B shows the relative position of cadambine molecules versus the center of mass of the S1 cavity along the simulation (black dashed line indicates the distance of the N3 inhibitor as reference). For panel C, size of the ribbon is proportional to the flexibility of the protein backbone and the size on the ligand center is proportional to the amount of structural deviation of the ligand during the 250 ns. Panel D reproduces the binding site S1 with the most important residues involved in the interactions. Figure S4: complete analysis of the 250 ns trajectory of 3CLpro of SARS-CoV-2bound with proanthocyanidin B2. Monomer A is shown in orange and monomer B in blue. Panels A, E, and F shows, from left to right, RMSD, all-to-all RMSD, PCA, and cluster counting (cutoff 1.5 Å). Panel B shows the relative position of proanthocyanidin B2 molecules versus the center of mass of the S1 cavity along the simulation (black dashed line indicates the distance of the N3 inhibitor as reference). For panel C, size of the ribbon is proportional to the flexibility of the protein backbone and the size on the ligand center is proportional to the amount of structural deviation of the ligand during the 250 ns. Panel D reprodu [file 4932572.f1.pdf]

Supplementary Materials

Investigating Potential Inhibitory Effect of *Uncaria tomentosa* (Cat`s claw) against the Main Protease $3CL^{Pro}$ of SARS-CoV-2 by Molecular Modeling

Andres F. Yepes-Pérez [1*], Oscar Herrera-Calderon [2*], José-Emilio Sánchez-Aparicio[3], Laura Tiessler-Sala[3], Jean-Didier Maréchal [3], Wilson Cardona-G[1]

1 Chemistry of Colombian Plants, Institute of Chemistry, Faculty of Exact and Natural Sciences University of Antioquia-UdeA, Calle 70 No. 52-21, A.A 1226, Medellin, Colombia; andresf.yepes@udea.edu.co (A.F.Y.-P.); wilson.cardona1@udea.edu.co (W.C.G.).

2 Academic Department of Pharmacology, Bromatology and Toxicology, Faculty of Pharmacy and Biochemistry, Universidad Nacional Mayor de San Marcos, Jr Puno 1002. Lima 15001, Peru; oherreraca@unmsm.edu.pe (O.H.-C.)

3 Insilichem, Departament de Química, Universitat Autònoma de Barcelona, Edifici C.n., 08193 Cerdanyola del Vallés, Barcelona, Spain; JeanDidier.Marechal@uab.cat (J.-D.M.); laura.tiessler@uab.cat (L.T.S.); joseemilio.sanchez@uab.cat (J.-E.S.-A.).

* Correspondence should be addressed to Andres F. Yepes-Pérez; andresf.yepes@udea.edu.co and Oscar Herrera-Calderon; oherreraca@unmsm.edu.pe

1. Supplementary figures

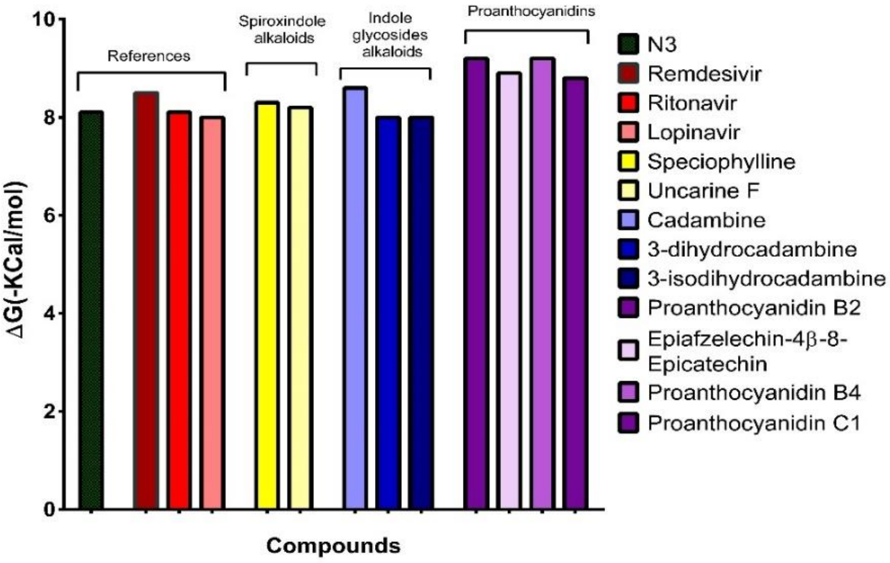

Figure S1. Histogram showing molecular docking results between the novel SARS-CoV-2 main protease and nine components from cat´s claw which showed the highest binding affinity.

spiroxindole alkaloids

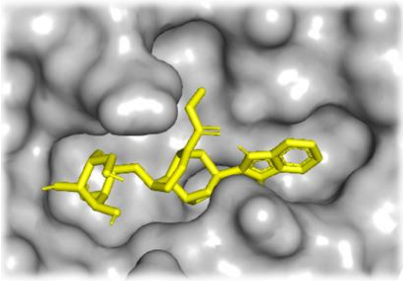

Speciophylline

ΔG= - 8.1 Kcal.mol^{-1}

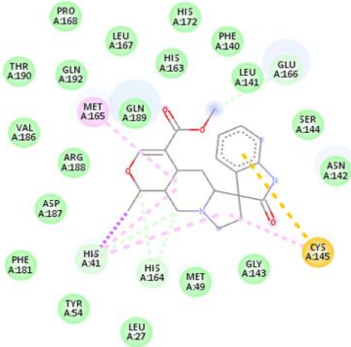

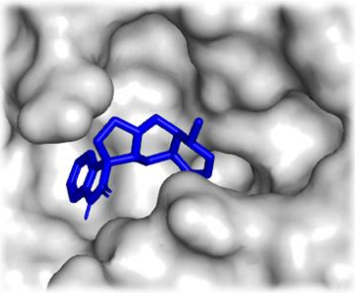

Uncarine F

ΔG= - 8.2 Kcal.mol^{-1}

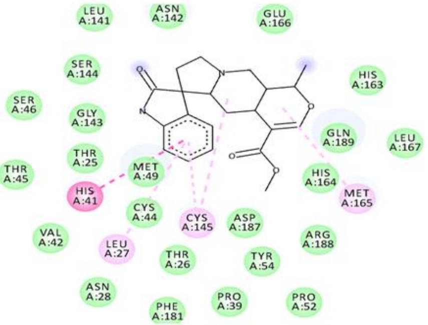

Indole glycosides alkaloids

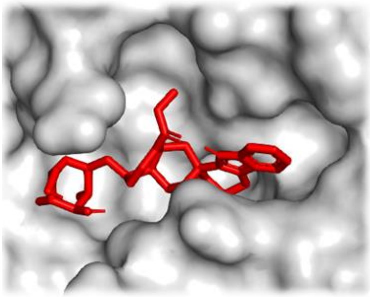

Cadambine

ΔG= - 8.6 Kcal.mol^{-1}

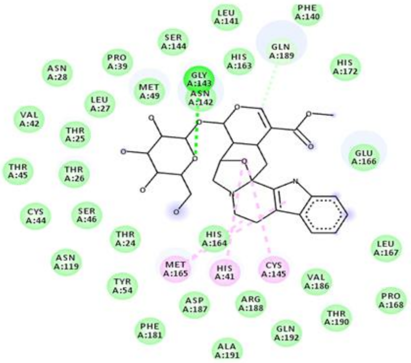

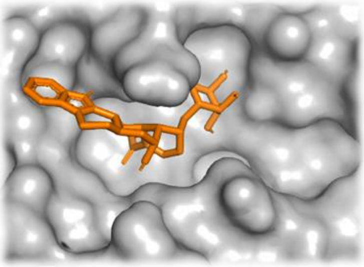

3-dihydrocadambine

ΔG= - 8.0 Kcal.mol^{-1}

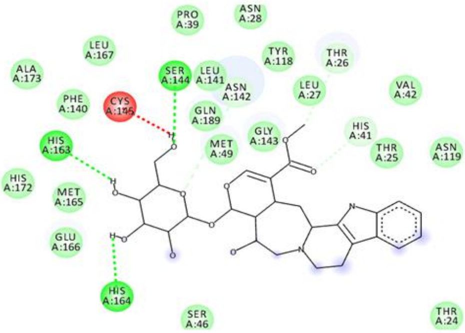

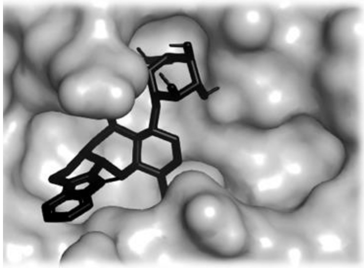

3-isodihydrocadambine

ΔG= - 8.0 Kcal.mol^{-1}

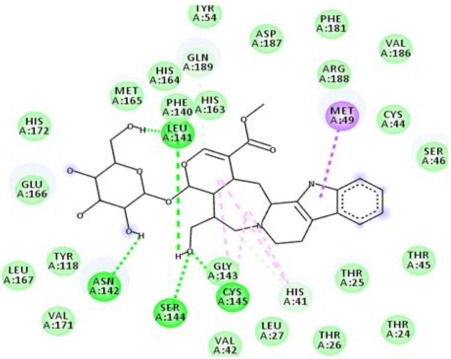

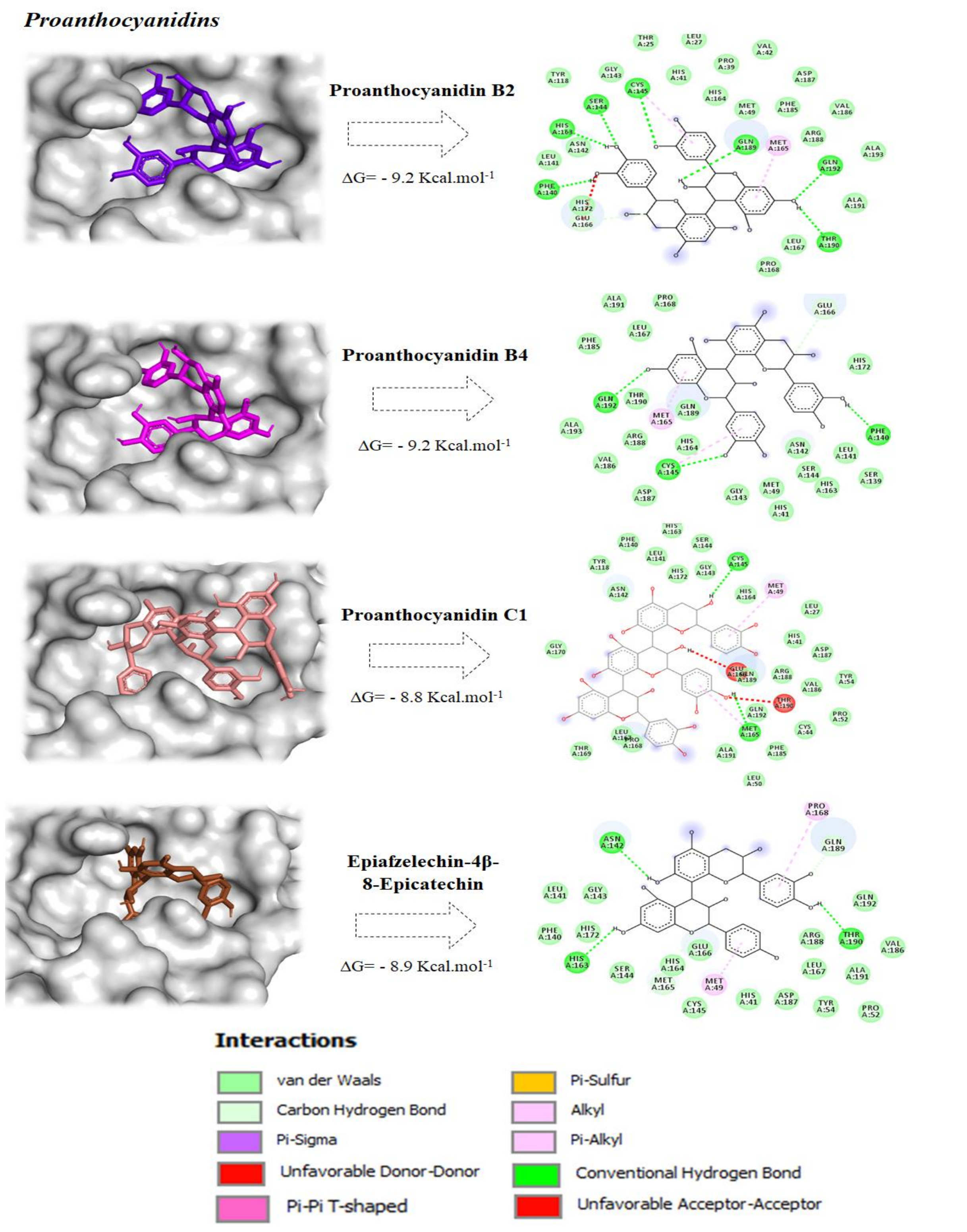

Figure S2. (A) The best conformation of the nine active components against 3CL^{Pro} within catalytic pocket of SARS-CoV-2 main protease 3CL^{Pro} (PDB: 6LU7). 2D interaction modes plots between the selected compounds with 3CL^{Pro}. Interactions between each component and amino acid residues into 3CL^{Pro} cleavage domain are indicated by the dashed lines.

Figure S2 illustrates 3D and 2D representations into the cleavage site on $3CL^{Pro}$ enzyme for the compounds which exhibited the highest docking score (Speciophylline, Uncarine F, Cadambine, 3-dihydrocadambine, 3-isodihydrocadambine, Proanthocyanidin B2, Epiafzelechin-4β-8-Epicatechin and Proanthocyanidin B4). We examined which interactions are involved when these compounds were docked against $3CL^{Pro}$ and how their structures affect them. Hence, those compounds having the better dock to protease on each series was selected for this analysis as follow: Speciophylline (for spiroxindoles series), Cadambine (for indole glycosides series) and Proanthocyanidin B2 (for Proanthocyanidins series). Thus, Speciophylline exhibits a good docking score for $3CL^{Pro}$ (−8.1 kcal/mol) when compared to the inhibitor N3. Speciophylline has π-π stacking interactions with CYS145 and MET165 residues. In addition, hydrophobic interactions between the molecule and the HIS164, HIS41, LEU141, GLN189, GLY143, GLU166 residues were observed. On the other hand, the docking score of Cadambine (−8.6 kcal/mol) was better than to the inhibitor N3. Cadambine showed a strong hydrogen bond interaction between the oxygen in pyran moiety with GLY143 residue. In addition, three π-stacking contacts were formed the molecule and the MET165, HIS41, CYS145 residues. Several hydrophobic interactions between Cadambine and MET49, GLU166, ASN142, GLN189 were also observed. Proanthocyanidin B2 was found to best bind with SARS-CoV-2 main protease (binding energy -9.2 kcal/mol) exhibiting highest $3CL^{Pro}$ affinity than inhibitor N3 or FDA approved antiviral drugs, respectively. Their high affinity is consequence of Proanthocyanidin B2 displayed seven hydrogen bonding interactions with key aminoacids CYS145, SER144, HIS163, PHE140, GLN189, GLN192 and THR190 into the catalytic domain of $3CL^{Pro}$, one π-stacking contact with MET165 and hydrophobic interactions formed between the molecule and ASN142, HIS172, GLU166, HIS41. As aforementioned, recent reports revealed the specific biological role of proanthocyanidins for the potential treatments of various antiviral infections.

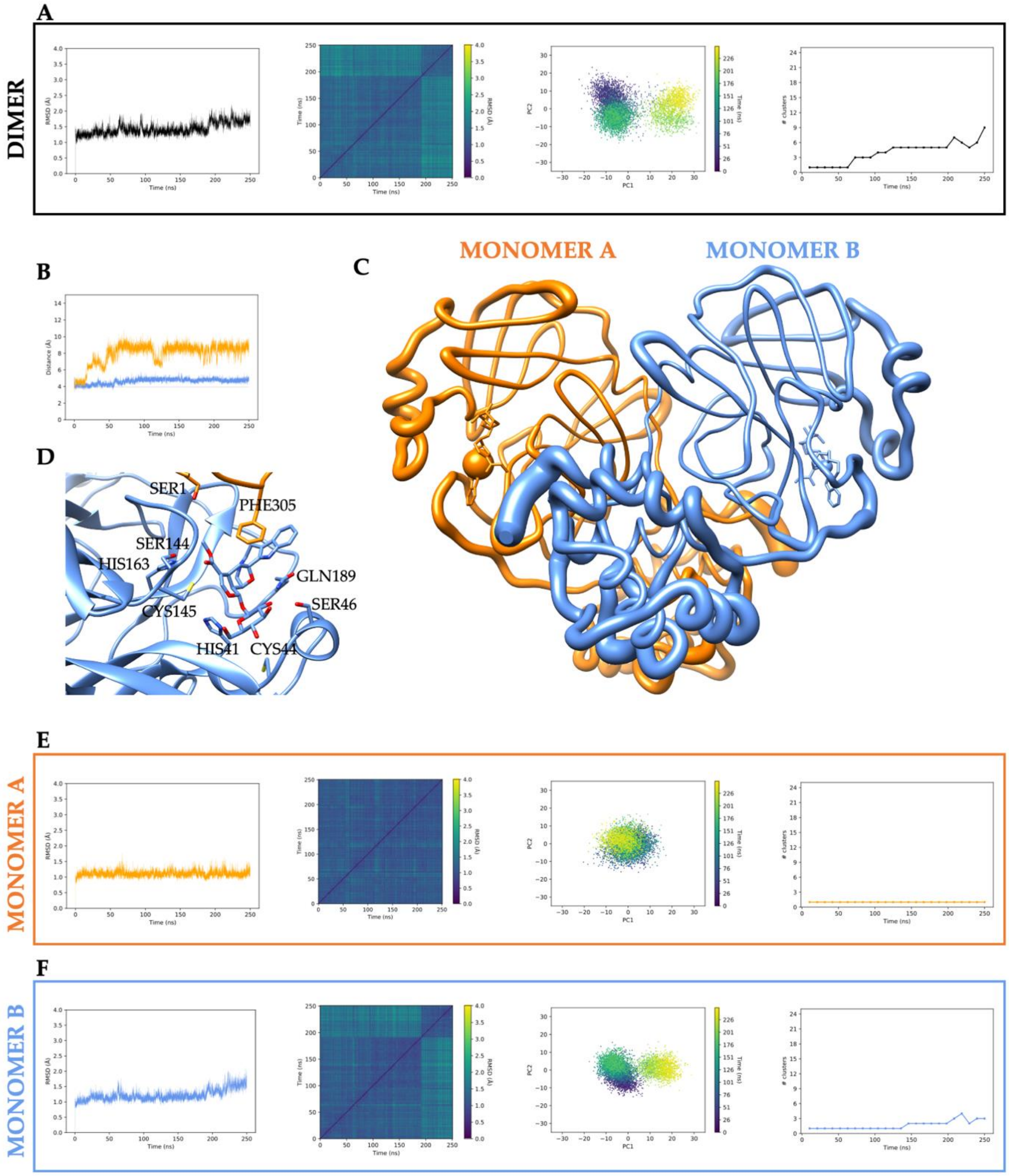

Figure S3. Complete analysis of the 250ns trajectory of $3CL^{Pro}$ of SARS-CoV-2 bound with Cadambine. Orange color reports to the monomer A and blue to monomer B. Panels A, E and F, contains, from left to right: RMSD, all-to-all RMSD, PCA and cluster counting (cutoff 1,5Å). Panel B shows the relative position of Cadambine molecules versus the center of mass of the S1 cavity along the simulation (black dashed line indicates the distance of N3 inhibitor as reference). For panel C, size of the ribbon is proportional to the flexibility of the protein backbone and the size on the ligand center is proportional to the amount of structural deviation of the ligand during the 250ns. Panel D reproduces the binding site S1 with the most important residues involved in the interactions.

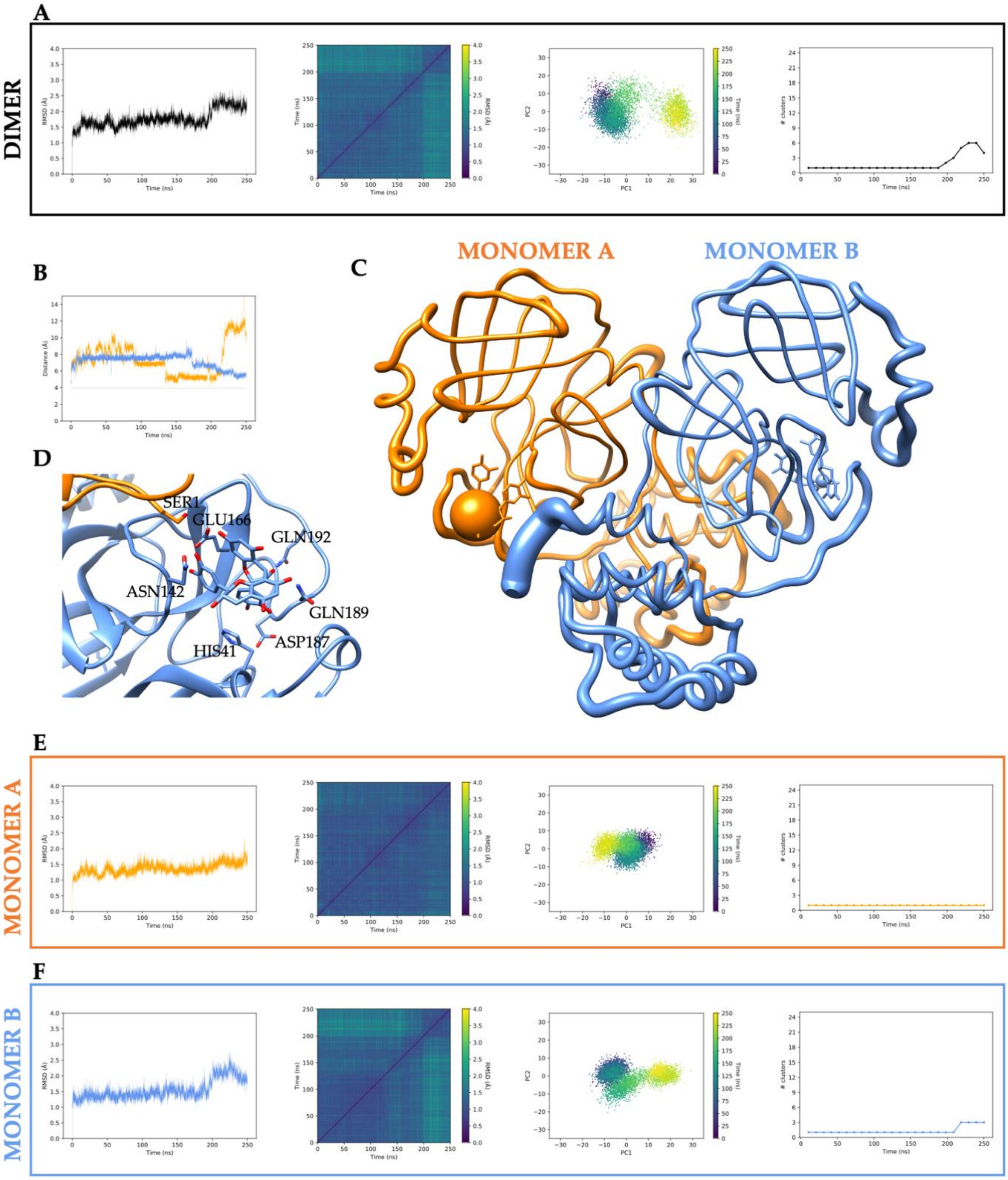

Figure S4. Complete analysis of the 250ns trajectory of 3CL^{Pro} of SARS-CoV-2 bound with Proanthocyanidin B2. Orange color reports to the monomer A and blue to monomer B. Panels A, E and F, contains, from left to right: RMSD, all-to-all RMSD, PCA and cluster counting (cutoff 1,5Å). Panel B shows the relative position of Proanthocyanidin B2 molecules versus the center of mass of the S1 cavity along the simulation (black dashed line indicates the distance of N3 inhibitor as reference). For panel C, size of the ribbon is proportional to the flexibility of the protein backbone and the size on the ligand center is proportional to the amount of structural deviation of the ligand during the 250ns. Panel D reproduces the binding site S1 with the most important residues involved in the interactions.

2. Supplementary tables

Table S1. GPathFinder vina barriers for the ligand route. 120 solutions were obtained for each ligand. Average and standard deviation are reported.

Ligand	Vina barrier average (kcal/mol)
Cadambine	4.31 ± 2.85
Proanthocyanidin B2	5.31 ± 3.17
Spheciophylline	2.38 ± 2.91

Table S2. Amino acids of the 3CL^{Pro} protease that participate in hydrogen bonding interactions with Cadambine, Proanthocyanidin B2 and Speciophylline.

	Cadambine	
**Time**	**Ligand 1 (Monomer A)**	**Ligand 2 (Monomer B)**
10ns	THR24, THR26, SER46, GLY143, SER144, CYS145, GLN189	THR26, GLY143, SER144, GLN189
50ns	GLU47, GLN189	THR26
100ns	GLU47, GLN189	THR26, CYS44, SER46, GLY143, SER144, GLN189
120ns	GLU47, GLN189	THR26, HIS41, CYS44, SER46, GLY143, SER144, GLN189
200ns	GLU47, GLN189	THR26, HIS41, CYS44, SER46, GLY143, SER144, GLN189
250ns	SER46, GLU47, GLN189	THR26, CYS44, GLY143, SER144, GLN189, GLN306

	Proanthocyanidin B2	
**Time**	**Ligand 1 (Monomer A)**	**Ligand 2 (Monomer B)**
20ns	SER46, GLU166	SER1, ASN142, GLU166, GLN189
50ns	SER46, GLU166, GLN189	SER1, ASN142, GLU166
75ns	SER46, GLU47, ASN142, GLU166, GLN189	SER1, GLU166
125ns	THR25, CYS145, HIS164, GLU166, GLN189	PHE140, GLU166, GLN189
165ns	SER46, MET49, LEU141, ASN142, SER144, CYS145, HIS164, GLU166, ASP187, GLN189	PHE140, GLU166
230ns	GLU166, GLN189	GLY143, HIS163, HIS163, HIS164, GLU166, ASP187, GLN189

	Speciophylline	
**Time**	**Ligand 1 (Monomer A)**	**Ligand 2 (Monomer B)**
20ns	GLN189, GLN192	GLN189, GLN192
45ns	GLN189, GLN192	GLN189, GLN192
80ns	ASN142, GLN192	GLN192
125ns	GLN189, GLN192	GLN192
165ns	GLN189, GLN192	ARG188, GLN189, THR190, GLN192
230ns	GLN189, THR190, GLN192	GLN189, GLN192

3. Input file (.yaml format) used in GpathFinder calculations


```yaml
ga:         # Section to configure the Genetic Algorithm parameters
    mut_pb: 0.8
    cx_pb: 0.2
    mut_indpb: 1
    generations: 750
    population: 12
genes:      # Section to configure the genes
-   module: gpath.genes.molecule
    name: Ligand
    path: ./structures/cadambine_pose01.mol2
-   module: gpath.genes.molecule
    name: Protein
    path: ./structures/protein
    first_frame: ./structures/protein/representative_1.pdb
-   module: gpath.genes.path_torsion
    name: T
    target: Ligand
    anchor: Ligand/3
-   module: gpath.genes.path
    name: Pathway
    ligand: Ligand
    protein: Protein
    torsion_gene: T
    min_step_increment: 0.5
    max_step_separation: 1.5
objectives:     # Section to configure the evaluation of the pathways
-   module: gpath.objectives.path_scoring
    name: Clashes
    probe: Pathway
    which: clashes
    method: average
    weight: -1.0
-   module: gpath.objectives.path_scoring
    name: Vina
    probe: Pathway
    which: vina
    method: average
    weight: -1.0
output:
    check_every: 0
    name: cadambine_pose01_00
    pareto: False
    path: ./cadambine_pose01_00
similarity:
    args:
    - Pathway
    - 1.0
    kwargs: {}
    module: gpath.path_similarity.pathways_rmsd

# Input file used with GPathFinder version 1.2.1
```
